# Methodological Comparison of Short-Read and Long-Read Sequencing Methods on Colorectal Cancer Samples

**DOI:** 10.3390/ijms26189254

**Published:** 2025-09-22

**Authors:** Nikolett Szakállas, Alexandra Kalmár, Kristóf Róbert Rada, Marianna Kucarov, Tamás Richárd Linkner, Barbara Kinga Barták, István Takács, Béla Molnár

**Affiliations:** 1 Department of Biological Physics, Faculty of Science, Eötvös Loránd University, 1117 Budapest, Hungary; szakallas.nikolett@semmelweis.hu; 2 Department of Internal Medicine and Oncology, Faculty of Medicine, Semmelweis University, 1083 Budapest, Hungary; kalmar.sopkez.alexandra@semmelweis.hu (A.K.); rada.kristof@hotmail.com (K.R.R.); tamas.linkner@3dhistech.com (T.R.L.); molnar.barbara.kinga@semmelweis.hu (B.K.B.); takacs.istvan@semmelweis.hu (I.T.); 3 Doctoral School of Applied Informatics and Applied Mathematics, BioTech Research Center, Óbuda University, 1034 Budapest, Hungary; kucarov.marianna@uni-obuda.hu; 4 3DHISTECH Ltd., 1141 Budapest, Hungary

**Keywords:** next-generation sequencing, short-read sequencing, long-read sequencing, colorectal cancer, methodology, comparative study

## Abstract

Colorectal cancer (CRC) is driven by a complex spectrum of somatic mutations and structural variants that contribute to tumor heterogeneity and therapy resistance. In this study, we performed a comparative analysis of short-read Illumina and long-read Nanopore sequencing technologies across multiple CRC sample groups, encompassing diverse tissue morphologies. Our evaluation included general base-level metrics—such as nucleotide ratios, sequence match rates, and coverage—as well as variant calling performance, including variant allele frequency (VAF) distributions and pathogenic mutation detection rates. Focusing on clinically relevant genes (*KRAS*, *BRAF*, *TP53*, *APC*, *PIK3CA*, and others), we characterized platform-specific detection profiles and completed the ground truth validation of somatic *KRAS* and *BRAF* mutations. Structural variant (SV) analysis revealed Nanopore’s enhanced ability to resolve large and complex rearrangements, with consistently high precision across SV types, though recall varied by variant class and size. To enable direct comparison with the Illumina exome panel, we applied an exonic position reference file. To assess the impact of depth and PCR amplification, we completed an additional high-coverage Nanopore sequencing run. This analysis confirmed that PCR-free protocols preserve methylation signals more accurately, reinforcing Nanopore’s utility for integrated genomic and epigenomic profiling. Together, these findings underscore the complementary strengths of short- and long-read sequencing platforms in high-resolution cancer genomics, and we highlight the importance of coverage normalization, epigenetic fidelity, and rigorous benchmarking in variant discovery.

## 1. Introduction

Recently, the application of the Illumina sequencing-by-synthesis technique has become more convenient in scientific communities compared to the first-generation Sanger and Maxam–Gilbert methods [1,2] due to its high throughput, cost and time efficiency, workflow and data output [3]. From the investigation of bacterial genomes to the precise identification of human oncomutations, many scientific publications demonstrate the outstanding performance of the method [4,5,6,7,8,9,10]. Generally, Illumina sequencing is based on the incorporation of fluorescently labeled nucleotides to the surface of a flow cell. The identification of nucleobases such as adenine (A), thymine (T), cytosine (C), and guanine (G) is facilitated by the different fluorescent expression profiles. The continuous development of the sequencing chemistry, instruments and bioinformatics analysis tools resulted in the fact that today, the overall efficiency of Illumina sequencing is approximately 99.99% [11,12]. The evolution of Illumina sequencing instruments began with the MiniSeq, followed by the MiSeq, and expanded to the HiSeq and NextSeq families. Although MiniSeq offers modest throughput of approximately 1.2 gigabases (Gb) per flow cell, HiSeq systems significantly increased capacity, supporting large-scale genome and transcriptome projects with output ranging from tens to hundreds of gigabases. The NextSeq series further advanced this scalability, delivering up to 500 Gb per flow cell depending on configuration. Methodologically, MiniSeq allows the sequencing of microbes and viruses, targeted sequencing based on amplicons, the targeted profiling of gene expression, analysis of miRNA and small RNA, and 16S metagenomic sequencing of the input samples. In contrast, HiSeq and NextSeq platforms support a broader range of high-throughput applications, including single-cell profiling (scRNA-Seq, scDNA-Seq, oligo tagging assays), transcriptome sequencing (total RNA-Seq, mRNA-Seq), DNA–protein interaction studies (ChIP-Seq), methylation sequencing, metagenomic profiling (shotgun metagenomics, metatranscriptomics), and cell-free DNA analysis for liquid biopsy diagnostics [13].

In addition to the obvious advantages and countless applications of Illumina sequencing, the problem of short reads and the necessary amplification steps prevent its exclusivity in practice. Simultaneously, the advent and advancement of long-read sequencing technology opened new avenues in the assembly of the bacterial, microbial, and human genomes as well as in methylation analyses involving nucleotide modifications beyond 5-methylcytosine [14,15,16,17,18,19]. The era of long-read sequencing also promoted the accurate identification of structural variants and repetitive elements at the whole-genome level [20,21]. One of the main representatives is Nanopore long-read sequencing, which exploits the power of pore-embedded motor proteins and ionic current to construct the nucleotide sequences. Over the years, the efficiency of Nanopore sequencing has continuously increased [19] and now theoretically approximates 99% accuracy. Although the method has great potential in clinical aspects, its high costs and several reported efficiency deviations and systematic uncertainties limit its success [22].

Previously, our work group performed a comprehensive study on whole-exome sequenced colorectal cancer samples. Kalmár et al. [23] investigated approximately 170 cases, using samples derived from patients confirmed with colorectal adenoma and carcinoma tumors, and also included the corresponding pairs of normal tissue associated with the tumor. Using this, we were able to examine the local mutation patterns of Hungarian colorectal cancer patients. This paper strongly supports the importance of Illumina sequencing in clinical settings. Furthermore, single-cell whole-exome sequencing of colorectal samples allowed us to uncover the heterogeneous nature of colorectal cancer. We selected both NAT and CRC samples from a patient and used laser microdissection to isolate 30 μm diameter regions from 12 distinct tissue locations. Whole-exome sequencing allowed us to characterize the mutational profiles of the samples and identify clinically actionable mutations relevant to targeted therapy [24]. Beyond these important findings, our goal was to expand our methodological knowledge and to take a step closer to whole-genome sequencing. Following the installation of a long-read sequencing device, we performed whole-genome sequencing on the same samples previously analyzed by Illumina whole-exome sequencing. This allowed us to directly compare the accuracy of short-read and long-read technologies and to extend our investigation of colorectal cancer to the whole-genome level. Building on the advantages and drawbacks of each approach, our objective was to provide a comprehensive comparison between short-read and long-read sequencing. Our analyses encompassed both whole-genomic and exonic DNA regions. Since consistent detection of genetic mutations depends heavily on sequencing read precision and coverage [25,26], as well as on variant characteristics, we also evaluated general read and base quality features.

## 2. Results

### 2.1. Data Preparation

In addition to the Illumina whole-exome data, we aimed to analyze the exonic regions within the Nanopore whole-genome sequencing reads. To accomplish this, we obtained predefined exome coordinates by downloading the BED file titled GRCh38 ILMN Exome 2.0 Plus Panel, which was developed by Twist Bioscience (San Francisco, CA, USA). This file contains three primary columns: the chromosome name, the start position, and the end position of each target region. Using this BED file, we filtered the Nanopore whole-genome alignment and variant data to include only the specified exonic regions. As a result, we compiled the following datasets for each sample: Original Illumina whole-exome data, original Nanopore whole-genome data and Nanopore exome data generated based on the Illumina exome panel.

### 2.2. The Mean Coverage Depth

The mean coverage values of the Nanopore whole-genome sequencing data for the healthy cancer-negative (NEG), tumor-associated normal matched (NAT) and colorectal cancer (CRC) samples were derived as 16.53X ± 9.86X, 27.82X ± 0.43X and 21.20X ± 6.60X, respectively. In contrast, by Illumina sequencing, we obtained 105.88X ± 30.34X, 102.92X ± 17.52X and 105.88X ± 35.18X depths over the target regions.

### 2.3. Quality Performance

The median mapping probabilities were analyzed to assess the base mapping quality (Phred) scores, which represents the measure of misaligned reads [27]. The mapping quality for all methods was higher than 20, which is equivalent to a mapping accuracy of 99%, at least. In more detail, the average mapping quality of the Nanopore method was 29.8, which equals an average precision of 99.89%. Consequently, the corresponding values were derived as 33.67 and 99.96% for the Illumina sequencing. The estimated average mapping errors were derived as 0.11% and 0.04% for the Nanopore and Illumina assays, respectively.

### 2.4. Nucleotide Content Analysis

**Figure 1:**
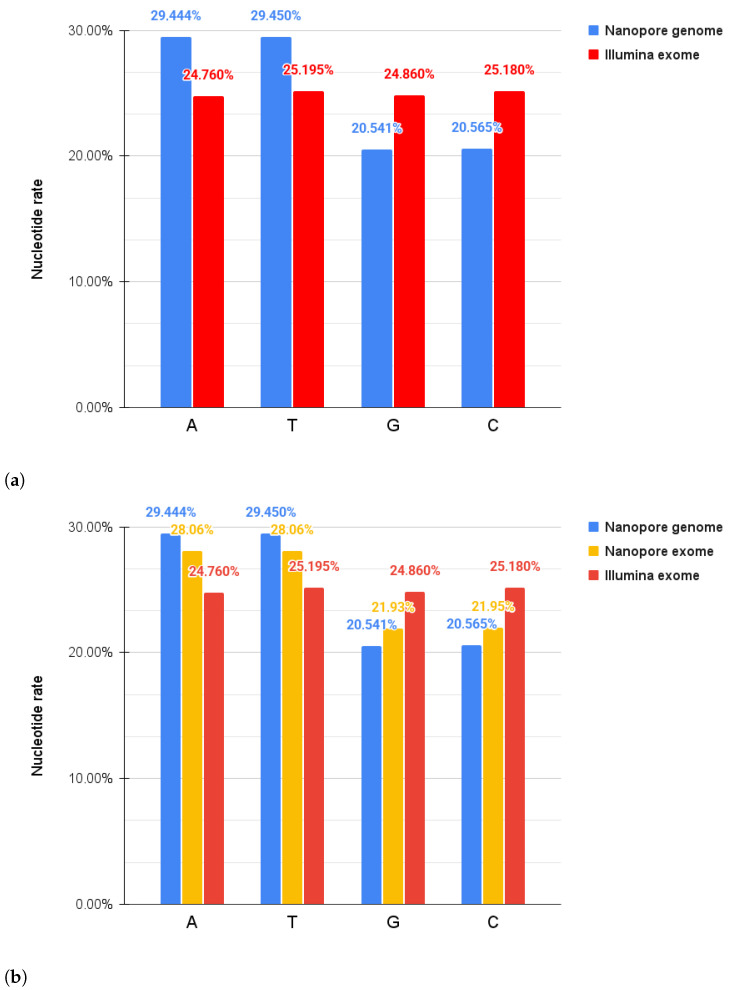
The distribution of A, T, G and C bases. (**a**) compares Nanopore whole-genome and Illumina whole-exome sequencing. (**b**) also includes the Nanopore exome results.

Our results showed that according to our expectations, the mean nucleotide content of the Nanopore DNA sequences derived was 29.444% ± 0.181% for As, 29.450% ± 0.179% for Ts, 20.541% ± 0.181% for Gs, and 20.565% ± 0.180% for Cs; while in the Illumina DNA sequence set, we calculated the nucleotide rate as 25.519% ± 0.580% for As, 25.654% ± 0.424% for Ts, 24.373% ± 0.418% for Gs, and 24.453% ± 0.561% for Cs, as shown in Figure 1a. To be exact, we expanded our analysis with the exome-restricted Nanopore whole-genome data and derived the following rates: A: 28.06% ± 0.20%, T: 28.06% ± 0.18%, G: 21.93% ± 0.19% and C: 21.95% ± 0.20%, and these can be seen in Figure 1b.

### 2.5. Targeted SNP Mutation Analysis in Exomes

Our research primarily focused on colorectal cancer-specific genetic mutations associated with resistance to therapy. Key genes under investigation included *TTN*, *APC*, *KRAS*, *TP53*, *PIK3CA*, *FBXW7*, *SOX9*, *SMAD4*, *SMAD2*, *NRAS*, *CTNNB1*, *GPC6*, *EGFR*, *BRAF*, *RNF43*, and *ARID1A* [28]. Through additional analysis using the KEGG pathway database, we found that many of these genes play critical roles in cancer-related signaling pathways, including PI3K-AKT, Ras, Wnt, TGF-beta, p53, ErbB, mTOR, and MAPK.

**Figure 2:**
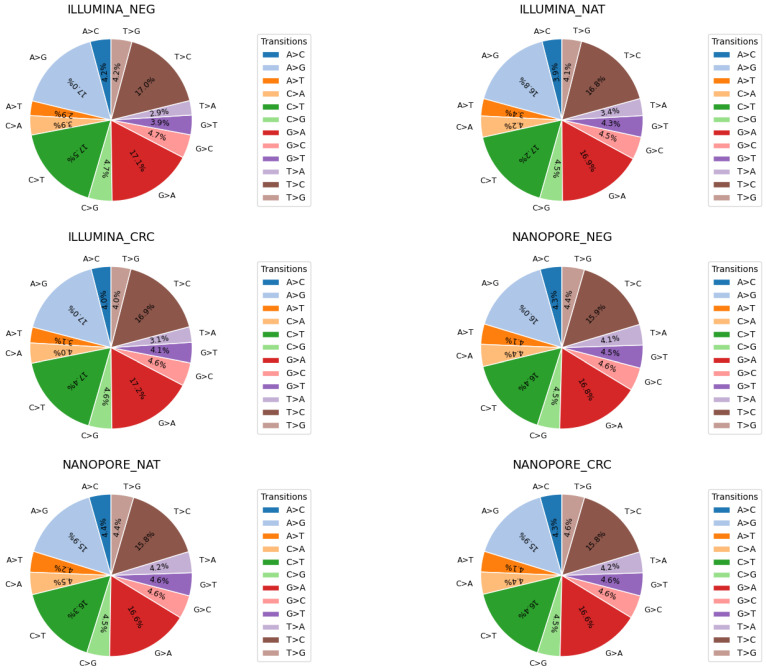
SNP transitions across all morphological groups using the results of Nanopore whole-genome and Illumina whole-exome sequencing analyses.

Mutational signatures are commonly defined by the patterns of base substitutions that occur within specific trinucleotide contexts. To explore this, we examined the distribution of single nucleotide polymorphism (SNP) transitions in all morphological groups (Figure 2). The corresponding pie charts illustrate the relative detection rates of twelve canonical base substitutions: A>C, A>G, A>T, C>A, C>G, C>T, G>A, G>C, G>T, T>A, T>C, and T>G. Our analysis revealed highly similar transition profiles between all groups, indicating that the observed mutational signatures are consistent and largely independent of the sequencing platform used.

**Figure 3:**
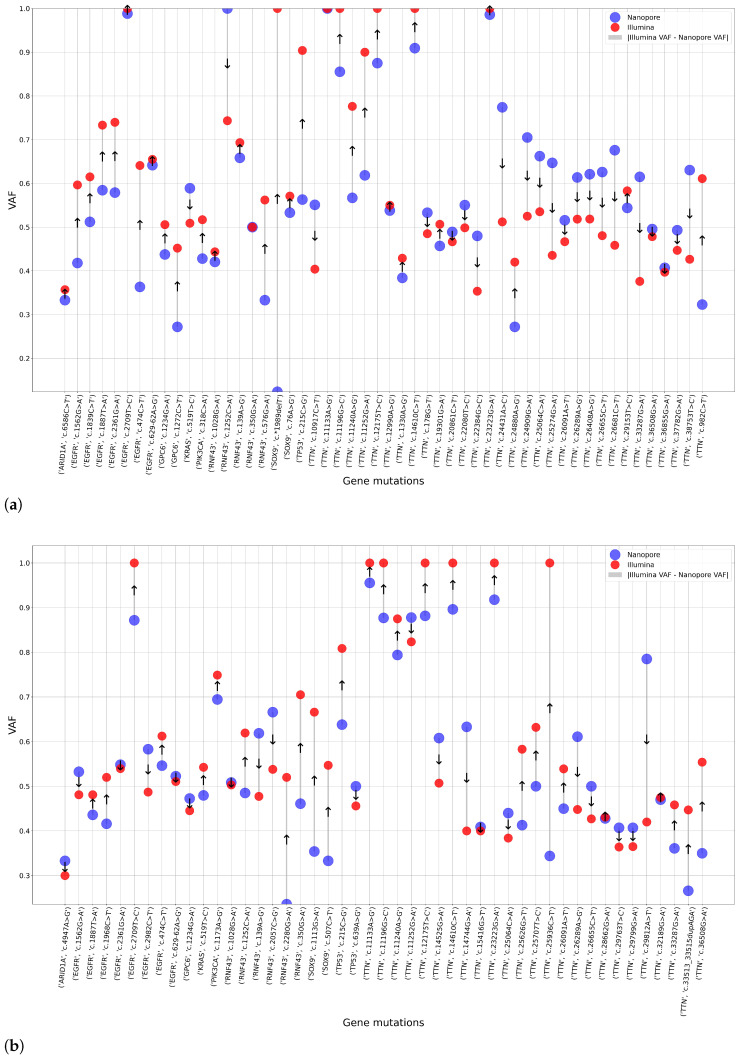

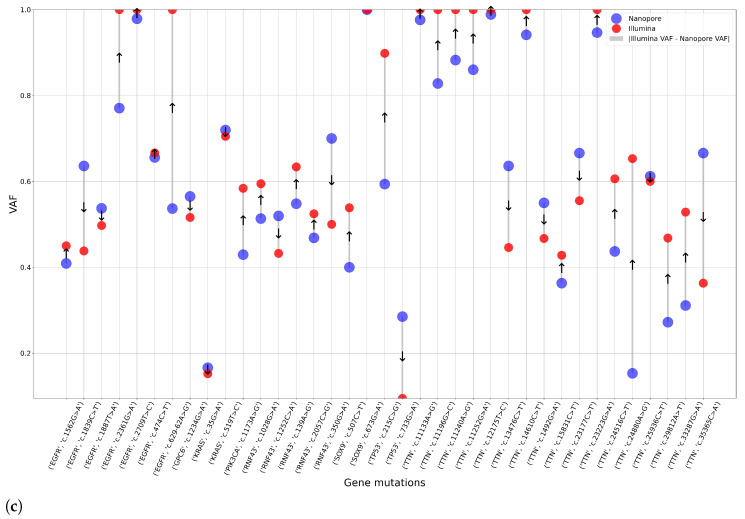
Variant allele frequency (VAF) values from Illumina exome panel restrictions using Nanopore and Illumina platforms. (**a**) displays results for the NEG group. (**b**) displays results for the NAT group, and (**c**) for CRC morphological classifications. The x-axis represents gene–mutation pairs, while the y-axis shows their corresponding VAF values. Blue circles indicate data from Nanopore sequencing, and red circles represent Illumina-derived values. Arrows indicate the direction of VAF differences: upward when Illumina VAF is greater than Nanopore, and downward when it is lower.

To assess the functional consequences of the identified mutations and to filter out those lacking clinical relevance, we retrieved colorectal cancer-specific mutation data from the COSMIC database [29], which was structured as a multicolumn dataset detailing key attributes such as gene name, CDS (coding sequence) identifier, and protein-level changes, and we developed a comprehensive database containing variant data for the aforementioned colorectal cancer-associated genes. After annotation, we qualitatively compared the two sequencing methods in the aspect of detected mutations. Then, we visualized the gene–mutation pairs identified by the Illumina and Nanopore sequencing methods within regions covered by the official Illumina Exome 2.0 Plus panel. These results are presented in Figure 3.

VAF is shaped by a variety of biological and technical factors. Although tumor heterogeneity and copy number alterations are known biological contributors, they are outside the scope of this study. Among technical variables, such as sequencing depth, read quality, mapping accuracy, library preparation bias, and platform-specific error profiles, we highlight the impact of coverage differences between Illumina and Nanopore, which significantly affect VAF uniformity. Read quality and mapping performance were consistently high across both platforms, allowing us to exclude these from further consideration. Library preparation bias, particularly PCR amplification artifacts, may affect Illumina data. Notably, platform-specific error patterns are as influential as coverage variation: Illumina is generally favored for accurate VAF quantification, especially in clinical or low-frequency variant contexts, while Nanopore excels in long-read sequencing and structural variant detection.

Seemingly, in this case, the distribution of individual variant allele frequencies (VAFs) appears non-normal and varies between morphological groups. In particular, no consistent pattern emerged to suggest the superior performance of one sequencing method over the other. Therefore, no statistically significant differences in mean VAF values can be established between the two platforms for mutations commonly detected within the Illumina exome panel target regions. However, our objective was to investigate low-VAF mutations separately on the sequencing platforms, and the results of this analysis will be presented in subsequent sections.

We further processed variant data derived from the restriction of original whole-genome nucleotide sequences to exome regions only and quantitatively summarized the results with respect to the number of detected mutations.

**Figure 4:**
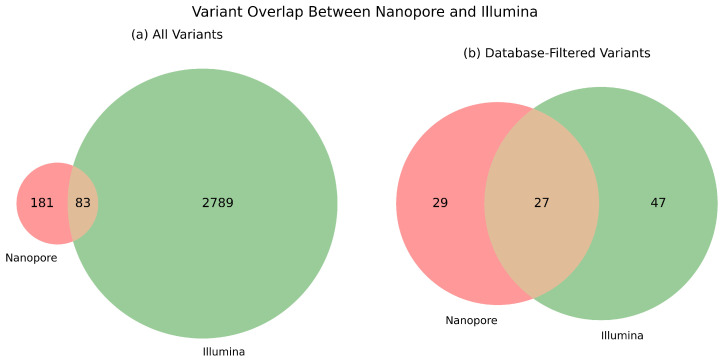
The number of detected mutations on the exonic region. All data are presented for Nanopore (N) and Illumina (I) sequencing. On the left side, results are illustrated to all variants, while on the right, only those are presented with clinically relevant characteristics.

**Figure 5:**
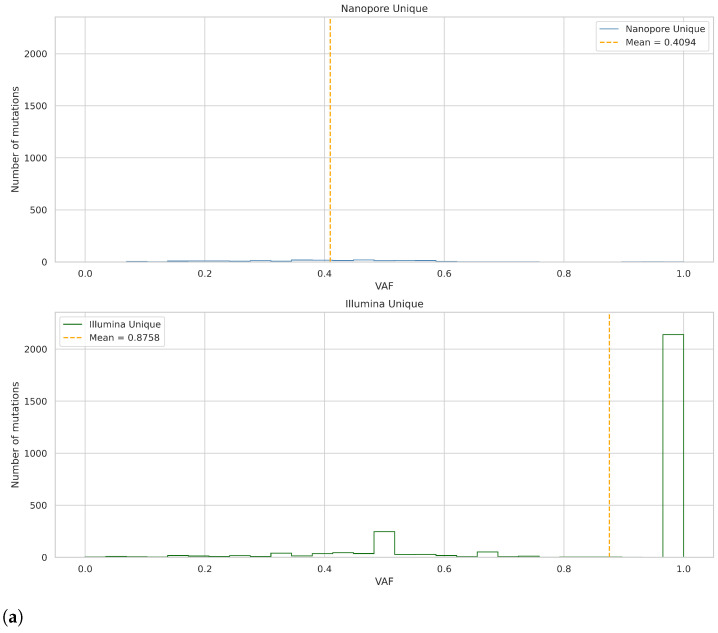

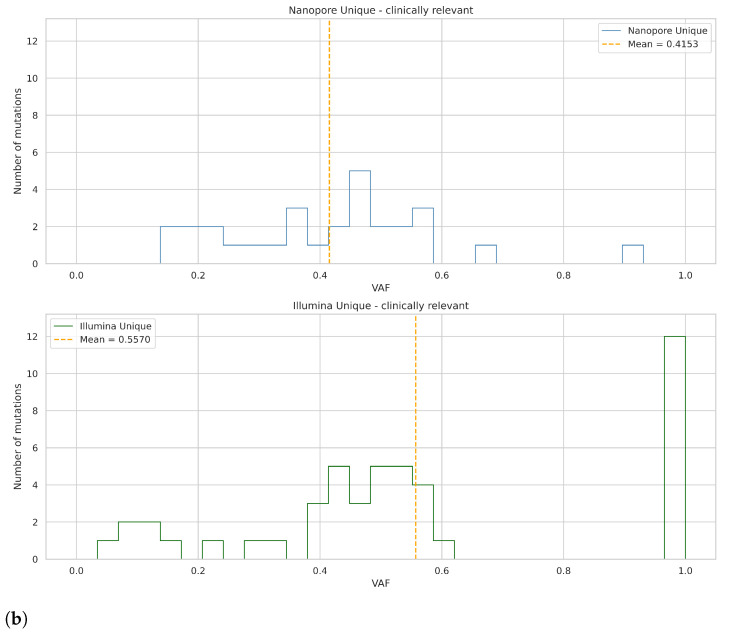
The VAF distribution of the commonly detected variants on the exonic regions of Nanopore and Illumina data. Results are presented to Nanopore and Illumina sequencing methods. In (**a**) one can see the VAF of all detected variants, while in (**b**), only the clinically relevant ones are visible.

Across all sample groups, we found 2789 method-specific mutations by Illumina and 181 by Nanopore sequencing, having a commonly detected pool of 83 variants. Regarding clinically relevant colorectal cancer genes, we detected 29 and 47 unique mutations by Illumina and Nanopore sequencing, respectively, and also 27 mutations were found to be common between the two sequencing approaches. This is summarized in Venn diagrams, which are presented in Figure 4. The distribution of the corresponding VAF values is illustrated in Figure 5.

The database-filtered comparison provides insight into the clinical utility and reliability of each platform to detect actionable mutations. The increased overlap in this panel suggests that both technologies are capable of capturing key driver mutations, though each may still miss certain database-confirmed variants due to platform-specific limitations.

### 2.6. Targeted SV Mutation Analysis in Exomes

**Figure 6:**
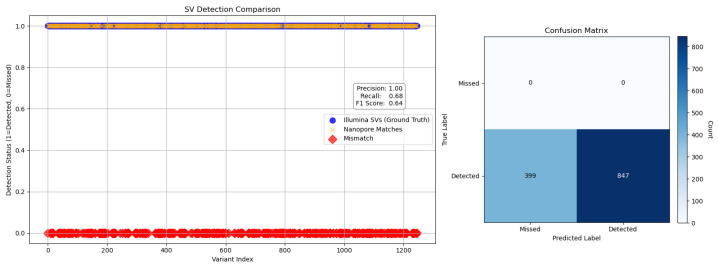
Structural variant (SV) detection performance comparison between Illumina (ground truth) and Nanopore sequencing. Left: A scatter plot showing detection status across variant indices. Blue circles represent SVs called by Illumina, yellow crosses indicate Nanopore-detected matches, and red diamonds mark mismatches (missed by Nanopore). Detection metrics are summarized in the inset: precision = 1.00, recall = 0.68, and F1 score = 0.64, indicating high specificity but moderate sensitivity. Right: Confusion matrix summarizing SV detection performance using Illumina as the reference. Of 1246 true SVs, Nanopore correctly detected 847 and missed 399, resulting in a recall of 0.68 and a precision of 1.00. This high precision, coupled with the upset plot’s large number of Nanopore-unique calls, suggests that many SVs detected by Nanopore may be true positives not captured by Illumina rather than false positives. Together, these figures illustrate both the quantitative detection performance and the qualitative breadth of SV discovery enabled by Nanopore sequencing.

**Figure 7:**
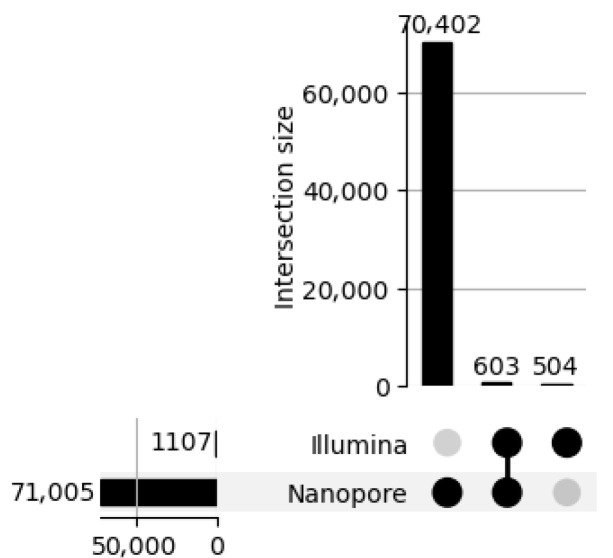
Structural variant (SV) overlap between Illumina and Nanopore sequencing platforms. The upset plot visualizes the intersection size of SV calls, highlighting the number of shared and unique variants. Nanopore detected approximately 70,402 SVs, while Illumina identified 1107 with 603 SVs shared between the two datasets. This substantial discrepancy reflects Nanopore’s enhanced sensitivity to large and complex SVs, which is consistent with its long-read capabilities. The dot matrix below the bar chart indicates membership across platforms with black dots marking shared SVs and gray dots marking platform-specific calls.

To evaluate the performance of Nanopore sequencing in the detection of structural variants (SVs), we performed a comparative analysis using Illumina-derived SV calls as a reference. Detection metrics, including precision, recall, F1 score, false positives and false negatives, were calculated to quantify concordance between platforms (Figure 6 and Figure 7), following the benchmarking framework proposed by Zook et al. [30], which emphasizes standardized evaluation across sequencing technologies for robust variant calling.

**Figure 8:**
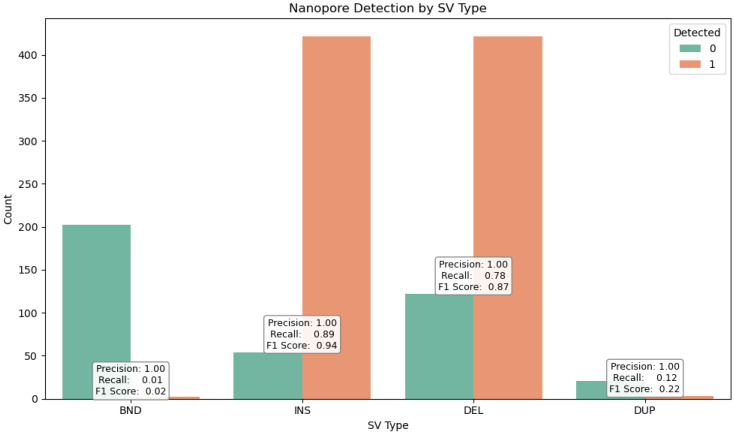
Detection performance of Nanopore sequencing across structural variant (SV) types. The bar chart displays the number of detected (orange) and undetected (green) SVs for each category: BND (breakend), INS (insertion), DEL (deletion), and DUP (duplication). Precision, recall, and F1 score values are shown above each SV type. While precision remains consistently high (1.00) across all types, recall varies significantly—ranging from 0.01 for BND to 0.89 for INS—indicating that Nanopore sequencing is highly specific but differentially sensitive depending on SV type. The high F1 score for insertions (0.94) and deletions (0.87) reflects strong overall performance for these classes, whereas duplications and breakends show limited recall and lower F1 scores.

**Figure 9:**
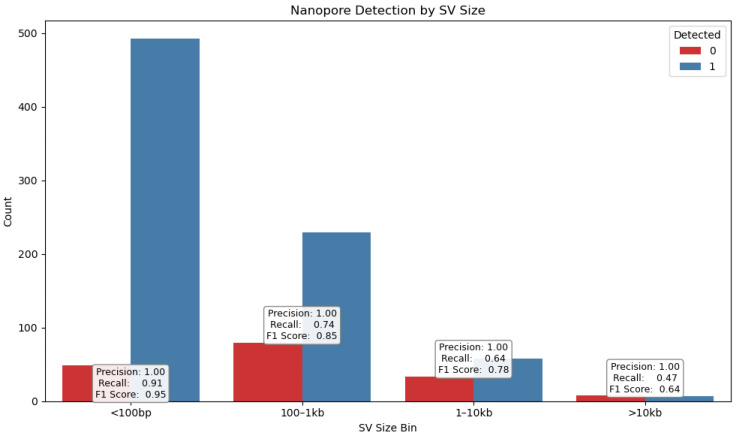
Detection performance of Nanopore sequencing across structural variant (SV) size categories. The bar chart displays the number of detected (blue) and undetected (red) SVs stratified by size bins: <100 bp, 100–1 kb, 1–10 kb, and >10 kb. Precision remains consistently high (1.00) across all bins, indicating that Nanopore calls are highly specific. However, recall decreases with increasing SV size—from 0.91 in the <100 bp bin to 0.47 in the >10 kb bin—resulting in a corresponding drop in F1 score from 0.95 to 0.64. These findings suggest that while Nanopore excels at detecting small and mid-sized SVs, its sensitivity for very large variants may be limited by current algorithmic or coverage constraints.

**Figure 10:**
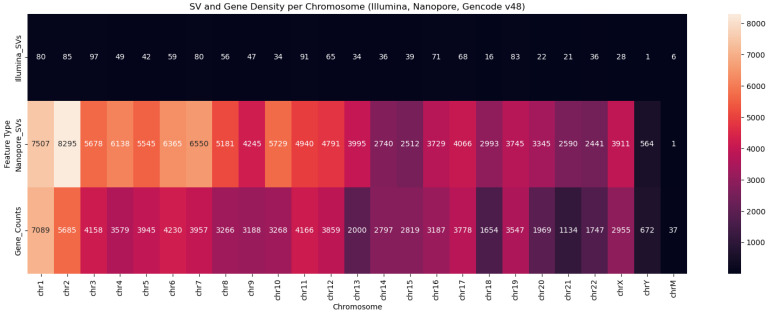
Chromosome-wide distribution of structural variants (SVs) and gene counts across Illumina, Nanopore, and Gencode v48 datasets. The heatmap visualizes the density of SVs detected by Illumina (top row) and Nanopore (middle row), alongside annotated gene counts from Gencode v48 (bottom row), across chromosomes chr1 to chrM. Nanopore shows markedly higher SV counts across all autosomes, with peak densities on chromosomes 2, 3, and 4, which is consistent with its enhanced sensitivity to large and complex variants. Illumina SV counts are comparatively sparse, reflecting platform limitations in resolving long-range genomic rearrangements. Gene density correlates with SV hotspots in several regions, notably chr1, chr2, and chr11, suggesting potential functional relevance. This visualization supports the conclusion that Nanopore sequencing provides broader SV coverage.

SVs were stratified by type (Figure 8)—deletions (DELs), duplications (DUPs), inversions (INVs), and insertions (INSs)—as well as by size (Figure 9), using defined bins (<100 bp, 100–1 kb, 1–10 kb, >10 kb). This stratification revealed distinct detection profiles (Figure 10) consistent with prior findings by Sedlazeck et al. [31], who demonstrated that long-read platforms such as Nanopore are particularly effective for identifying complex SVs like insertions and inversions.

**Figure 11:**
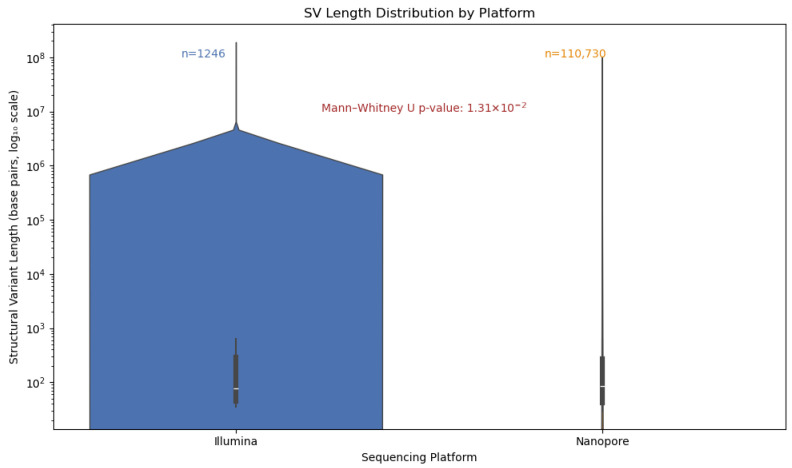
Structural variant (SV) length distribution across sequencing platforms. The violin plot compares the distribution of SV lengths detected by Illumina and Nanopore sequencing with the y-axis scaled logarithmically to accommodate the wide range of variant sizes. Illumina-derived SVs (n = 1246) exhibit a narrower distribution concentrated around shorter lengths, while Nanopore-derived SVs (n = 110,730) span a broader range, including substantially longer variants. Despite Nanopore’s higher SV count, its violin appears visually thinner due to the dispersed nature of its length distribution, resulting in lower density at any specific range. A Mann–Whitney U test confirms a statistically significant difference in SV length distributions between platforms (*p* = 1.31 × 10^−2^), supporting that Nanopore sequencing enables more comprehensive detection of long and complex structural variants.

Given the known limitations of short-read technologies in resolving large or complex variants, we hypothesized that Nanopore sequencing would exhibit a broader and more diverse SV length spectrum. We visualized these distributions using violin plots to capture both the density of SVs across length ranges (Figure 11). This approach enabled us to disentangle the detection breadth from the detection density, revealing that while Illumina SVs are tightly clustered around shorter lengths, Nanopore detects a substantially larger number of SVs spanning a wider range, including long variants exceeding 10 kb. These findings underscore the enhanced resolution and sensitivity of long-read sequencing for complete SV discovery.

### 2.7. Validation Methods

Using Illumina whole-exome sequencing, we identified multiple oncogenic mutations with potential therapeutic relevance. Among the five samples of colorectal cancer (CRC) analyzed, three exhibited alterations in the *KRAS* gene: CRC3 contained a substitution for G12D, CRC5 showed a G12S mutation, and CRC4 carried a Q61H variant. Furthermore, sample CRC2 harbored a V600E mutation in the *BRAF* gene. In particular, these mutations could not be reliably detected using Nanopore sequencing technology.

To validate the accuracy of our sequencing findings, we supplemented our analysis with droplet digital PCR (ddPCR) data and available clinical test results. The ddPCR analysis was performed similarly as completed in the study of Kalmár et al. [23]. Using the ddPCRTM KRAS G12/G13 Screening Kit, we independently assessed the *KRAS* mutations in the relevant CRC samples. We also reviewed diagnostic records to gather mutation data where accessible. This approach confirmed the presence of G12D and G12S mutations in CRC3 and CRC5, respectively, and verified *BRAF* Mutation V600E in CRC2 through clinical tests. For certain samples, clinical documentation or mutation-specific assays were not available. A comprehensive summary of these results is provided in Table 1.

### 2.8. High-Coverage Sequencing

**Figure A1:**
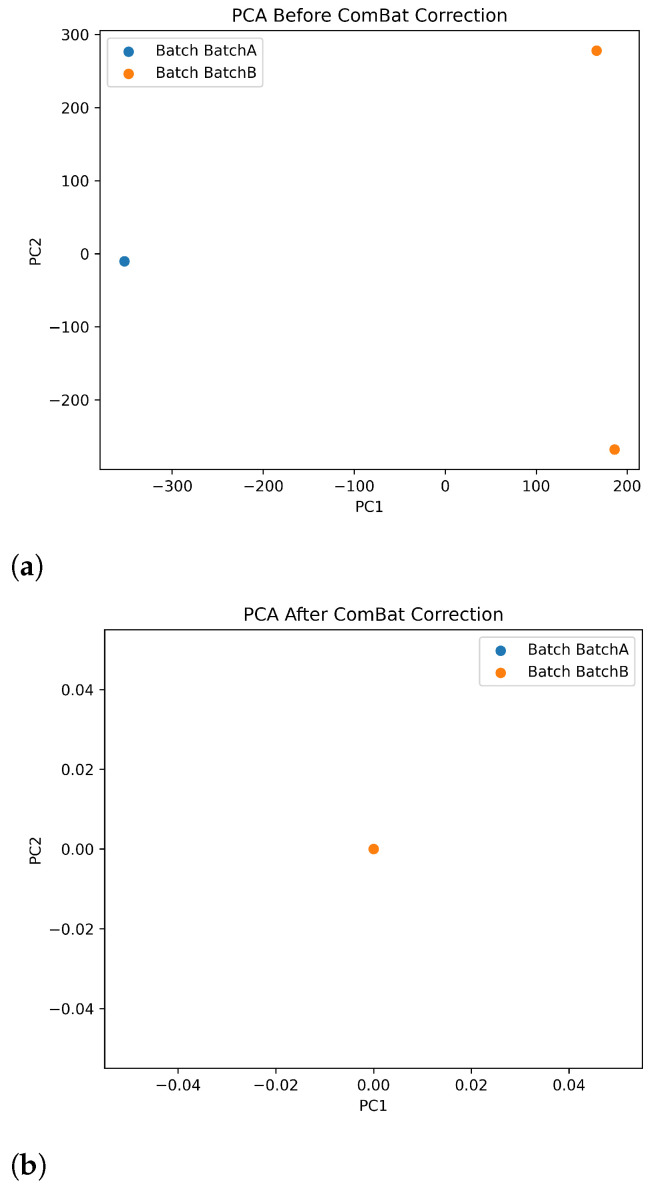
Principal component analysis (PCA) of imputed genotype data colored by sequencing batch. Prior to batch correction, (**a**) samples from the first run (BatchA, blue) and second run (BatchB, orange) form distinct clusters along PC1 and PC2, indicating a strong batch effect. After ComBat correction, (**b**) the batch-specific clustering is eliminated, with both batches overlapping near the origin, suggesting the successful mitigation of batch effects. PC1 and PC2 together explain 100% of the variance.

The successful detection of pathogenic variants is highly dependent on the coverage of the sequencing [32]. To prove this, we chose two of our colorectal cancer samples to repeat the experiments with higher coverage. As we were satisfied with the original Illumina sequencing coverage of the samples in question, it was not necessary to replicate the short-read experiment. For this end, we completed the Nanopore protocol using two additional flow cells per sample. By combining the original and the new data, we were able to reach 70X-90X coverages. To ensure that our results were not confounded by technical variation, we also assessed potential batch effects between sequencing runs. Details of the metrics compared, PCA analysis, and ComBat correction are provided in the Appendix A (Table A1, Figure A1a,b).

**Figure 12:**
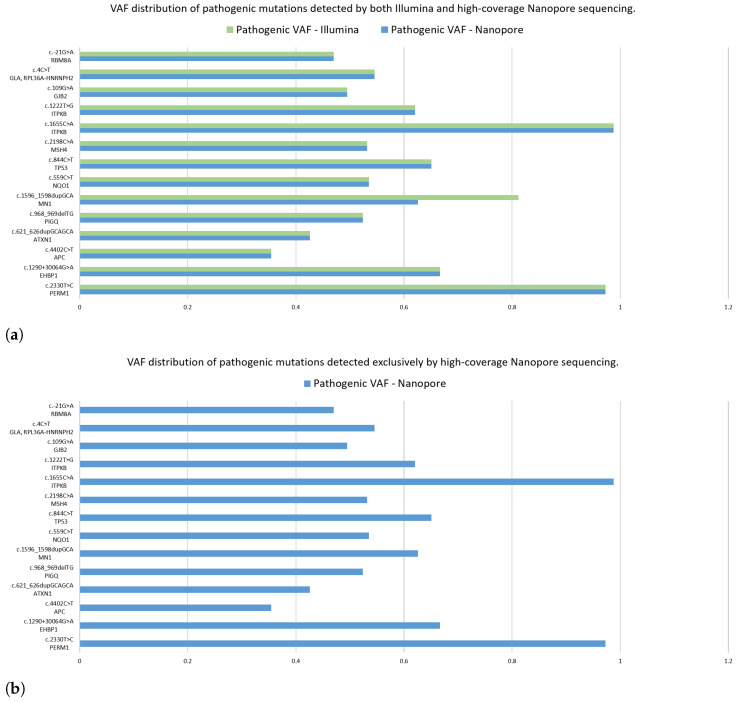
The VAF distribution of the pathogenic mutations detected (**a**) by both Illumina (green) and high-coverage Nanopore (blue) sequencing, and only Nanopore high-coverage sequencing (**b**). The vertical axis represents the ID of mutations and the corresponding genes, while on the horizontal axis, VAF values are shown.

After the completion of high-coverage sequencing, we compared ClinVar-annotated mutations detected by Nanopore and Illumina assays. Following this, we analyzed the data of the two Nanopore high-coverage and the corresponding Illumina CRC samples. Our findings revealed that of the 316 and 72 detected mutations, 210 and 26 were in clinically relevant colorectal cancer genes, for Nanopore and Illumina sequencing, respectively. Further restricting our criteria, variants with pathogenic/likely pathogenic clinical significance occurred, and 14 of them were detected by both methods, while Nanopore sequencing was able to detect 16 more. To support our findings, we included the VAF data of the mutations. Figure 12a shows the VAF values for mutations detected simultaneously by the two assays. Taking into account the pathogenic alterations identified by only Nanopore sequencing, Figure 12b aimed to illustrate these gene mutations and their VAF rate.

With a particular focus on genes *TTN*, *APC*, *KRAS*, *TP53*, *PIK3CA*, *FBXW7*, *SOX9*, *SMAD4*, *SMAD2*, *NRAS*, *CTNNB1*, *GPC6*, *EGFR*, *BRAF*, *RNF43* and *ARID1A*, we identified pathogenic variants in the *APC* (c.4402C>T) and *TP53* (c.844C>T) genes using both sequencing platforms.

### 2.9. The Effect of PCR Amplification on Sequencing Results

Encouraged by the fact that we experienced discrepancies in the occurring rate of nucleotide bases and in variant detection results, we aimed to test a hypothesis which assumes that perhaps the absence/presence of PCR amplification during Nanopore/Illumina sequencing has an effect on these characteristics. Thus, we completed an additional Nanopore experiment in which the samples were prepared involving a PCR amplification step. Particular care was taken to ensure that the resulting data achieved sufficiently high coverage across the targeted regions.

**Figure 13:**
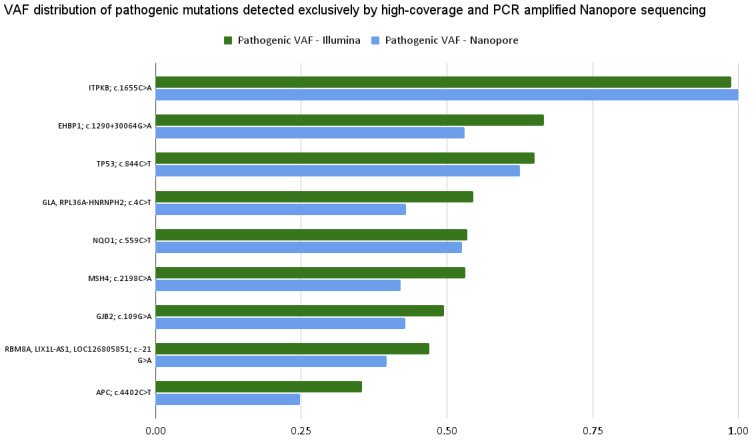
The VAF distribution of the detected mutations when PCR amplification was involved during Nanopore sequencing.

The first result of this additional analysis showed that PCR amplification has no effect on nucleotide occurrence rates. For all bases, these values remained almost the same as they were in the case of original Nanopore whole-genome sequencing: A: 29.74% ± 0.01%, T: 29.94% ± 0.02%, G: 20.08% ± 0.04%, C: 20.22% ± 0.03%. Regarding the variant detection performance, we can also conclude that PCR amplification has no effect on it, not even its absence/presence during Nanopore, and its presence during Illumina sequencing. We were able to detect every mutation by both assays, as illustrated in Figure 13. Furthermore, since we performed high-coverage Nanopore sequencing, similar to the previous high-coverage results, we detected many more Nanopore-specific mutations.

As is well known, PCR amplification greatly affects modified bases 5mC and 5hmC because the methylation patterns are not maintained during amplification. To verify this, we performed a methylation analysis on the PCR amplified Nanopore data and summarized our results in Table A2. As expected, the proportion of 5mC and 5hmC decreased.

### 2.10. Low-Frequency Variant Detection

To evaluate the precision and sensitivity of Nanopore versus Illumina sequencing in detecting low-frequency somatic mutations, we performed a comparative analysis using matched datasets in three sample groups: NEG, NAT, and CRC. Variant calls were extracted from VCF files, filtered to include only mutations in 16 genes associated with colorectal cancer, and stratified by platform (Nanopore vs. Illumina). We applied platform-specific logic to extract variant allele frequencies (VAFs) and removed duplicate mutations based on gene and CDS identity. The variants were then classified into three groups: Nanopore-only, Illumina-only, and Common to both platforms. To assess low-frequency detection, we binned VAFs into intervals, counted the number of variants per bin for each platform, and compared VAF distributions between the three variant categories. Our analysis indicates that Nanopore sequencing underperforms relative to Illumina in detecting low-frequency variants, including clinically significant pathogenic mutations, highlighting limitations in its sensitivity for rare but important alterations.

**Figure 14:**
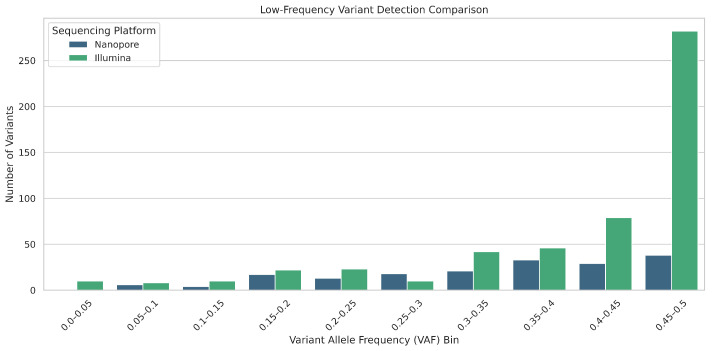
Venn diagrams showing the overlap of detected mutations between Nanopore and Illumina platforms both before and after filtering against a curated mutation database.

As demonstrated in Figure 14. Illumina consistently detected more variants in the lower VAF bins (especially <0.15), indicating higher sensitivity to low-frequency mutations. Nanopore showed a steep increase in detection only in higher VAF bins (>0.3) with the highest count in the 0.45–0.5 range. This supports the concern that Nanopore may underperform in detecting low-VAF mutations, which are common in heterogeneous tumor samples.

**Figure 15:**
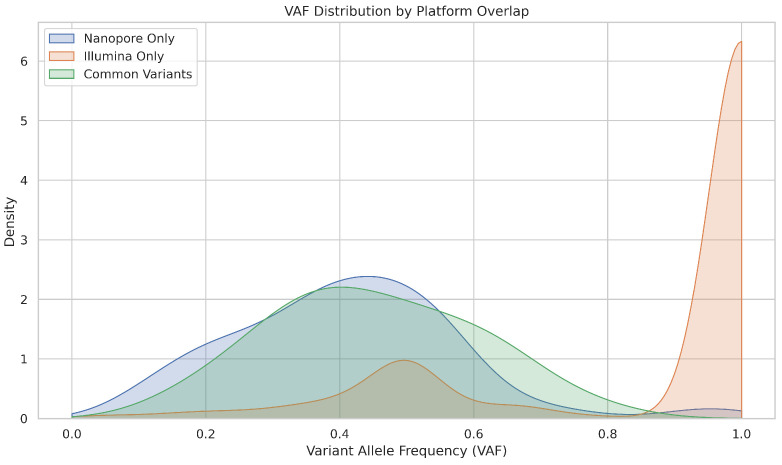
Comparison of variant allele frequency (VAF) distributions across Nanopore-only, Illumina-only, and commonly detected mutations, highlighting platform-specific sensitivity to low-frequency variants.

Figure 15 illustrates that Illumina-only variants showed a sharp peak near VAF = 1.0, suggesting strong detection of clonal mutations and possibly overcalling high-frequency events. The Nanopore-only variants had a broader distribution but fewer low-VAF calls. Common variants were distributed over a moderate VAF range, indicating reliable detection by both platforms for mid-frequency mutations.

In summary, we can conclude that Illumina is more sensitive to low-frequency variants, making it better suited to detect subclonal mutations. Nanopore, while capable of reliably detecting high-VAF mutations, may miss low-VAF events due to its higher systematic error rate. The overlap between platforms is strongest in mid to high VAF ranges, suggesting that clinically relevant mutations with moderate allele frequencies are robustly detected by both technologies.

To specifically assess the performance of Nanopore sequencing in detecting pathogenic *KRAS* mutations at codons 12, 13, and 61, we performed an intra-platform comparison across three Nanopore datasets: high coverage, original, and PCR-amplified. Despite this targeted focus, none of these clinically relevant *KRAS* variants were detected in any of the Nanopore datasets, as reflected in the validation results. All datasets were derived from the same sample cohort and filtered to include only *KRAS* mutations with variant allele frequencies (VAFs) categorized using a threshold of 0.3 to distinguish low-frequency variants from those with higher allelic representation.

**Figure 16:**
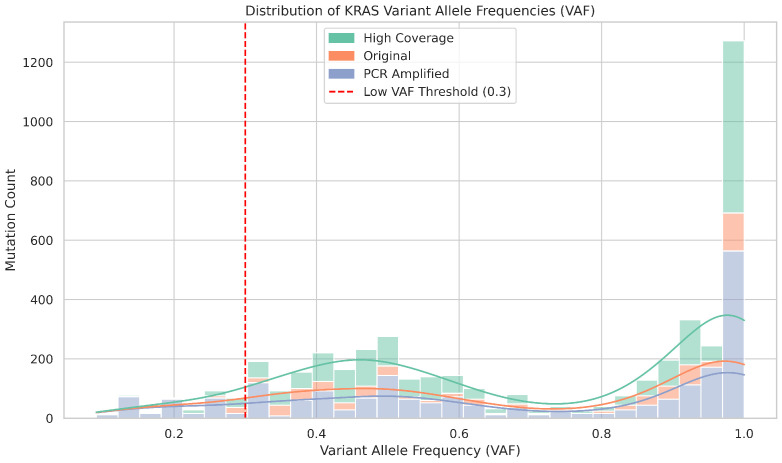
Distribution of KRAS variant allele frequencies (VAF) across three Nanopore sequencing strategies. Histogram showing the frequency of KRAS mutations by VAF in high coverage, original, and PCR-amplified Nanopore datasets. A vertical dashed line at VAF = 0.3 marks the threshold used to distinguish low-frequency variants. High coverage data show a concentrated peak of high-VAF mutations with limited low-VAF detection, while PCR-amplified data display a broader distribution, including a higher number of low-VAF events.

As shown in Figure 16, the distribution of VAFs reveals distinct patterns in the three sequencing strategies. The high coverage dataset exhibited a concentrated peak of high-VAF mutations, with a modest tail of low-frequency events, suggesting improved sensitivity while maintaining specificity. The original dataset showed fewer total mutations and minimal detection of low-VAF variants, which was consistent with a reduced sequencing depth. In contrast, the PCR-amplified dataset showed a broader VAF distribution and a noticeably higher count of low- and high-frequency *KRAS* mutations, indicating increased detection capacity that may be influenced by amplification bias.

**Figure 17:**
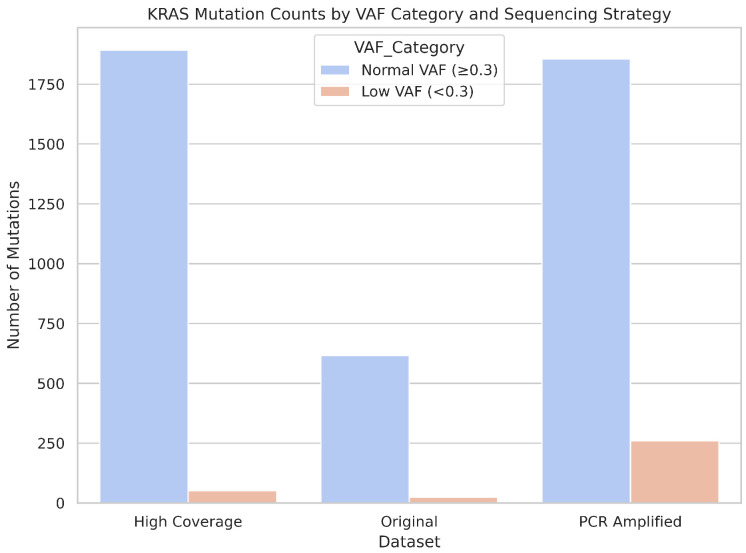
KRAS mutation counts stratified by VAF category and sequencing strategy. Bar chart comparing the number of KRAS mutations with low VAF (<0.3) and normal VAF (≥0.3) across high coverage, original, and PCR-amplified Nanopore datasets. PCR amplification yields the highest count of low-VAF mutations, whereas high coverage sequencing detects the most total mutations but remains conservative in calling low-frequency events.

Figure 17 further illustrates the breakdown of *KRAS* mutation counts by VAF category. Although high coverage sequencing yielded the highest number of total mutations, it remained conservative in calling low-VAF events. However, the PCR-amplified dataset demonstrated a marked increase in the detection of low-VAF mutations, suggesting that amplification may enhance the sensitivity to subclonal variants, albeit with potential trade-offs in specificity.

Together, these results highlight the influence of sequencing depth and amplification on the detection of *KRAS* mutations within Nanopore workflows. While high coverage improves the overall yield of mutations and can capture some low-frequency events, and PCR amplification may further increase sensitivity for rare variants, pathogenic KRAS mutations at codons 12, 13, and 61 remained undetected. These findings underscore the importance of optimizing sequencing protocols based on the clinical or research context, particularly when detecting subclonal or clinically critical mutations.

## 3. Discussion

Despite the extensive literature on sequencing technologies [33,34], few studies offer direct methodological comparisons between short-read and long-read platforms in a clinical context. Our study addresses this gap by evaluating performance in multiple genomic dimensions in colorectal cancer samples. As everyone seeks assurances about the participation of sequencing methods in everyday diagnostics—which can be a groundbreaking milestone—we must first analyze their common and individual strengths in detail [35,36]. In this study, our objective was to thoroughly investigate the base and variant level performance of short and long-read sequencing to provide their characteristics over the results [37,38].

As the first step of our investigation, we summarized the general quality, coverage, base and variant statistics of the long-read and short-read results [39,40]. Subsequently, we applied the Illumina manufactured exome panel [41] to restrict data from the whole genome sequence to exonic regions. Although adaptive sequencing methods are available for which region selection is carried out in parallel with the sequencing process [42,43], due to the participation of samples in other whole genome sequencing studies, we subsequently completed these selections using bioinformatic methods [44].

Regarding the four main DNA nucleotide bases, A, T, G and C, we expected that their distribution and rate in the genome and exome would be different [45,46,47]. The average GC content in a whole-genome is around 41%, while for whole-exome sequences, it can increase to 52%. The base composition analysis revealed that while the genomic GC content is around 40% (A, T = 20% and G, C = 30%), in the exome, it increases to 50%, showing an even distribution between bases with rates of 25%–25%–25%–25%.

Analysis of single nucleotide polymorphism (SNP) transitions revealed that the sequencing platform—whether short-read or long-read—did not significantly influence overall mutational signatures [48,49]. This suggests that intrinsic mutational processes dominate over platform-specific biases in base substitution patterns. Through targeted variant assessment within exonic regions, we observed that the VAFs of mutually detected mutations were largely independent of both tissue morphology classifications and sequencing methodologies [38,50].

When evaluating the combined set of unique and shared variants, Illumina sequencing identified a higher total number of mutations compared to Nanopore sequencing under standard (non-high-coverage) conditions [51]. This is consistent with Illumina’s higher base accuracy and depth, which favor the sensitive detection of low-frequency variants. However, when clinically relevant mutations were specifically considered, those with functional or diagnostic significance, Nanopore exhibited performance comparable to that of Illumina [52,53]. In particular, Illumina detected 47 unique variants, Nanopore identified 29 unique variants, and both platforms shared a common subset of 27 mutations. These findings suggest that while Illumina may offer broader coverage, Nanopore sequencing is also sufficiently reliable for clinical variant profiling, especially when rapid turnaround or long-read context is prioritized [15].

To address the precision and sensitivity of Nanopore versus Illumina sequencing in detecting low-frequency somatic mutations, we performed a comparative analysis using matched datasets, categorizing variants by tissue morphology and detection characteristics. Variant allele frequencies (VAFs) were stratified into defined intervals to assess platform-specific performance. Illumina demonstrated superior sensitivity for low-frequency variants (<0.15 VAF), making it well suited for subclonal mutation detection. Nanopore sequencing, while less sensitive in this range, reliably identified high-VAF mutations and offered broader structural variant coverage. Furthermore, Nanopore and Illumina also exhibited a sharp peak near VAF = 1.0, potentially reflecting an overcalling of high-frequency variants. Nanopore variants displayed a broader VAF distribution, with fewer low-frequency calls, which was likely attributable to their higher intrinsic error rate. In general, these findings support the conclusion that Illumina is more sensitive to low-VAF mutations and may be better suited to detect subclonal alterations in complex tumor landscapes.

To evaluate the structural variant (SV) detection performance of Nanopore sequencing, we benchmarked it against Illumina-derived SV calls. Nanopore achieved exceptionally high precision (1.00) across all SV types and size categories, reflecting strong specificity, while recall was more variable, with an overall value of 0.68 and an F1 score of 0.64, indicating moderate sensitivity. Its long-read capability enabled the detection of complex and large SVs (>10 kb) that were largely missed by Illumina, resulting in the identification of 70,402 SVs compared to 1107 by Illumina, with only 603 shared between platforms. Length-based stratification further highlighted platform-specific strengths: Nanopore excels at large SV detection, particularly insertions and deletions, whereas short SVs (<100 bp) are more prone to misclassification or omission due to sequencing noise and limited resolution. SV density mapping across chromosomes 2, 3, and 11, as well as upset plot visualization, underscored Nanopore’s enhanced ability to resolve complex rearrangements in gene-dense regions. Collectively, these findings demonstrate the utility of Nanopore sequencing for comprehensive SV detection and emphasize the importance of stratified evaluation, resolution metrics, and functional annotation in structural variant research, highlighting its complementary strengths to short-read platforms in cancer genomics.

Given the critical role of sequencing depth in effective variant detection, we performed high-coverage Nanopore sequencing on two colorectal cancer (CRC) samples [54,55]. After restricting the sequencing data to exonic regions, we compared variant profiles with those obtained from Illumina exome sequencing. Elevated coverage substantially improved Nanopore’s detection power, reflected in a higher total number of identified variants compared to Illumina [50,56], which was consistent with prior reports that long-read platforms benefit from deeper coverage, particularly in low-complexity or repetitive regions [50].

When filtering for clinically relevant variants, Nanopore continued to outperform Illumina in capturing additional variants. However, focusing specifically on pathogenic *KRAS* mutations at codons 12, 13, and 61, none were detected in any Nanopore dataset, highlighting a current limitation in sensitivity for these critical low-frequency mutations. For other clinically relevant alterations, both platforms identified the same set of pathogenic variants associated with CRC, namely *APC* (c.4402C>T) and *TP53* (c.844C>T), underscoring concordance in detecting disease-specific variants [56,57]. This observation emphasizes that while Nanopore sequencing offers advantages for comprehensive variant discovery, additional optimization may be required to reliably capture key pathogenic point mutations in genes like *KRAS*.

To ensure the integrity of our variant data and account for possible non-biological variation, we systematically evaluated batch effects arising from differences in sequencing timing and flow cell usage. Although all samples were processed under standardized laboratory conditions by the same technician, one sample was sequenced several months prior to the others, prompting a two-part assessment. First, we compared key sequencing metrics, including coverage, nucleotide base composition, and mapping quality, among flow cell batches to identify discrepancies in technical performance. Second, we performed principal component analysis (PCA) on the variant data to visualize the variation related to the batch. The PCA revealed a clear separation between BatchA and BatchB along the first two principal components, indicating that technical factors, rather than biological differences, were driving the observed variation. To correct for this confounding effect, we applied the ComBat algorithm, which effectively harmonized the data between batches while preserving the underlying biological signal. Post-correction PCA confirmed the elimination of the batch-driven structure, resulting in a unified distribution of samples. These findings underscore the importance of rigorous batch effect assessment and correction in high-throughput sequencing studies, particularly when samples are processed at different time points or under experimental conditions.

Finally, we assessed the impact of incorporating PCR amplification during Nanopore sequencing. Our comparative analysis revealed that PCR amplification did not significantly alter the overall nucleotide composition, as base occurrence frequencies remained consistent with those observed in the original whole-genome Nanopore data. Although PCR amplification had minimal impact on base composition and variant detection, it significantly disrupted methylation profiling, highlighting the importance of PCR-free protocols for epigenetic studies.

However, the implications for epigenetic analysis are more profound. It is well established that PCR amplification disrupts the detection of modified bases, particularly 5-methylcytosine (5mC) and 5-hydroxymethylcytosine (5hmC), due to the inability of DNA polymerases to preserve methylation marks during amplification [58,59]. To validate this effect, we performed methylation profiling on PCR-amplified Nanopore reads. As anticipated, the relative abundance of 5mC and 5hmC was reduced to near-background levels, confirming the loss of modified base information attributable to PCR-induced biases [60,61]. These findings underscore the necessity of PCR-free workflows for accurate epigenetic characterization using Nanopore sequencing [62].

Although cost remains a barrier to widespread Nanopore adoption, its unique strengths—such as direct methylation detection and comprehensive SV profiling—position it as a valuable complement to short-read platforms in precision oncology. As such, the widespread adoption of Nanopore for routine clinical use may require further reductions in cost or strategic implementation in scenarios where its distinct advantages outweigh financial considerations.

## 4. Conclusions

In conclusion, our comparative analysis highlights the complementary strengths of Illumina and Nanopore sequencing technologies in colorectal cancer genomics. Illumina remains the gold standard for detecting low-frequency variants due to its high base-level accuracy, making it particularly suitable for clinical diagnostics and subclonal mutation profiling. Nanopore sequencing, in contrast, excels in detecting structural variants, preserving epigenetic information, and providing comprehensive genome coverage, especially when sequencing depth is increased.

Although high-coverage Nanopore sequencing enhances the overall variant detection sensitivity, we observed that certain clinically relevant pathogenic *KRAS* mutations at codons 12, 13, and 61 were not detected, underscoring current limitations in detecting low-frequency point mutations. Nevertheless, Nanopore’s ability to capture complex genomic rearrangements and methylation signals positions it as a powerful tool for integrated genomic and epigenomic profiling, complementing short-read platforms.

Together, these sequencing approaches provide a synergistic strategy for comprehensive molecular characterization. Their strategic integration holds promise for more precise, scalable, and personalized cancer diagnostics, enabling both high-resolution variant detection and broader genomic context assessment.

## 5. Materials and Methods

### 5.1. Clinical Samples

Together, in this study, we involved five NEG samples, five NAT samples, and five CRC samples. All samples were obtained after receiving the written informed consent of the untreated patients. Colonic specimens were collected during surgery from tumors and histologically normal adjacent tissue (NAT) at the 1st Department of Surgery of Semmelweis University, Budapest, Hungary. The samples were then stored at −80 °C until use. In addition, tissue samples from the same locations were immediately fixed in buffered formalin, and experienced pathologists established the histological diagnoses. The study was carried out according to the Declaration of Helsinki and was approved by the local ethics committee and the government authorities (Regional and Institutional Committee on Science and Research Ethics (ETT TUKEB) Nr.: 14383-2/2017/EKU Semmelweis University, Budapest, Hungary).

### 5.2. Whole-Exome DNA Isolation

The whole exome DNA isolation, the preparation of the whole exome library and the next-generation sequencing process were carried out similarly to that written in the oncogenomic study by Kalmár et al. [23].

### 5.3. Whole-Exome Bioinformatic Analysis

The demultiplexing and FASTQ file generation was performed using the Illumina BaseSpace [63] interface. We used FastQC and MultiQC (v.3.9.5) tools to assess the quality of sequencing reads. The raw sequence reads were aligned with the Human Reference Genome GRCh38. SNP and short indel germline and somatic variants were called and determined by the Dragen Germline Variant Caller v.4.2.4 (Illumina Inc., San Diego, CA, USA) on NEG, CRC, and NAT samples. Dragen Somatic Variant Caller v.4.2.7 (Illumina Inc.) was run in tumor-normal mode for CRC and NAT samples. In the next steps, the vcf variant call files were annotated using the SnpEff eff variant annotation tool, and vcf2maf generated the maf mutation annotation files [64]. The clinical impact of the variants was evaluated according to the ClinVar [65] database. Mutation characteristics were summarized using the maftools [66] ‘plotmafsummary’ tool (v.3.21).

### 5.4. Specific Bioinformatic Analysis of Sequences Restricted to Exome Regions

The generation of mutation annotation databases for all samples was followed by the investigation of mutational profiles compared to the Catalog Of Somatic Mutations In Cancer (COSMIC) [29] restricted to genetic variations of colorectal adenocarcinoma. It was completed using a self-made Python (v.3.13.7) script, where we loaded the unique mutational and COSMIC datasets and performed comparative investigations. The results were illustrated by packages matplotlib [67] and seaborn [68]. In the next step, we compared the long-read and short-read sequencing data with a focus on shared and diverse exonic mutations. The outcome of this analysis was presented on scatterplots where the x and y axes represented the single nucleotide polymorphisms of colorectal cancer-specific genes and the number of mutations on them, respectively. The goal of this analysis was to present the individual performance of different sequencing methods. The transitions of SNPs were demonstrated on pie charts where the number and fraction of unique base shifts are marked. We conducted investigations on highly mutated genes and illustrated them by denoting the number of their mutations on the x-axis, while the name of the genes was on the y-axis. The variant summary plots were generated by maftools regarding the different groups for both sequencing methods.

### 5.5. Whole-Genome DNA Isolation

The homogenization of solid tissue samples was completed using the MagNA Lyser instrument in Tissue Lysis Buffer and with MagNA Lyser Green Beads Tubes (Roche Diagnostics GmbH, Manheim, Germany). Proteinase K (4 mg/μL; Roche Diagnostics GmbH) digestion was carried out at 56 °C for 2 h, which was followed by total gDNA isolation using the High Pure PCR Template Preparation Kit (Roche Diagnostics GmbH) according to the manufacturer’s instructions. The protocol also included an RNA elimination step using RNase A/T1 mix (2 mg/mL of RNase A and 5000 U/mL of RNase T1; ThermoFisher Scientific, Vilnius, Lithuania) for 1 h at 37 °C. DNA was eluted in 100 μL ultrapure water (Rnase- and Dnase-free) and stored at −20 °C until further use. To measure sample concentration, we used the Qubit 1.0 fluorometer and the Qubit dsDNA HS and ssDNA Assay Kits (Invitrogen, Waltham, MA, USA). The purity of gDNA was confirmed on the NanoDrop fluorometer. Fragment length analysis of up to 60 kb was performed on the TapeStation 4200 system (Agilent, CA, USA) using the DNA ScreenTape assay.

### 5.6. Whole-Genome Library Preparation

Libraries were prepared using the Ligation Sequencing Kit V14 (SQK-LSK114, Oxford Nanopore Technologies Ltd., Oxford, UK) and the NEBNext Companion Module (New England Biolabs Ltd., Ipswich, MA, USA) according to the manufacturer’s instructions. Briefly, for each sample, 1 μg of genomic DNA (gDNA) was used as input and subjected to repair and final preparation with 1 μL of DNA CS (control strand) at 20 °C for 10 min, which was followed by 65 °C for 10 min. The magnetic bead was then cleaned using AMPure XP beads (Beckman Coulter Inc., Brea, CA, United States) in a 1:1 volume ratio (60 μL). After two wash steps, DNA samples were resuspended in 61 μL of nuclease-free water. DNA concentration was measured using the Qubit dsDNA HS Assay Kit on a Qubit 1.0 fluorometer (Invitrogen, Waltham, MA, USA). Adapter ligation was carried out for 10 min, which was followed by another AMPure XP bead cleaning using a 1:2.5 ratio (40 μL beads to 100 μL solution). The mixture was incubated on a Hula mixer for 5 min. The beads were washed with Short Fragment Buffer (SFB) and resuspended after each step. After incubation in 25 μL of prewarmed elution buffer for 10 min at 37 °C, the adapter-ligated gDNA was carefully pipetted off the beads. DNA concentration was measured again. The amount of gDNA input was calculated using an average fragment length of 8 kbp and an average base molar weight of 324.5, resulting in 35 fmoles. Flow cells (R10.4.1 DNA chips, Oxford Nanopore) were equilibrated at room temperature for approximately 30 min; then, they were inserted into the PromethION24 sequencer (Oxford Nanopore Technologies Ltd., Oxford, UK) and primed. The libraries were prepared in a final volume of 200 μL and thoroughly resuspended before loading. Sequencing was initiated immediately using simplex base calling and ran for 72 h.

### 5.7. Whole-Genome Bioinformatic Analysis

After 72 h of sequencing, we had the data in pod5 file format. Base calling was performed by a Dorado basecaller (Oxford Nanopore Technologies Ltd., Oxford, United Kingdom) with version 0.6.1. Raw sequence reads were aligned to the GRCh38 Human Reference Genome during basecalling. As we were interested in modified DNA bases, we used the Dorado configuration model dna_r10.4.1_e8.2_400bps_hac@v4.2.0@5mCG_5hmCG@v2 at the 4 kHz data sampling frequency. Sequencing metrics were calculated using the PycoQC [69] tool v2.5.2. The aligned reads were then sorted and indexed using the index and sort commands of the Samtools package (v.1.20) [70]. The short nucleotide polymorphism, structural variant, and copy number variations were called and annotated on the sorted and indexed aligned files by the wf-human-variation workflow v1.9.2 (Oxford Nanopore Technologies Ltd., Oxford, UK) with a minimum bam coverage of 9×. Furthermore, the genomic region of variant calling was restricted to exonic positions of the Illumina Exome 2.0 Plus Panel. The restriction was necessary to be able to align the variant call results of the short-read Illumina and long-read Nanopore sequencing methods in later steps. The modified bases were summarized using the modkit package v0.3.1 (Oxford Nanopore Technologies Ltd., Oxford, UK). The mutation annotation maf files were generated by the package vcf2maf [64] and the Ensembl Variant Effect Predictor (VEP) release with version 102 [71]. The clinical impact of the variants was evaluated according to the ClinVar database.

### 5.8. Generative AI

AI-assisted tools were used to support technical scripting and formatting tasks. Generative AI was not used to produce original scientific content or interpret experimental results.

## Figures and Tables

**Table 1:** Summary of KRAS G12/G13 findings and other relevant mutations.

KRAS G12/G13 Findings				
Sample	Mutation	Illumina	Nanopore	ddPCR Confirmation
CRC1	KRAS G12, G13	Wild type	Wild type	Yes
CRC2	KRAS G12, G13	Wild type	Wild type	Yes
CRC3	KRAS G12, G13	p.G12D, VAF = 15%	Wild type	Yes, VAF = 13%
CRC4	KRAS G12, G13	Wild type	Wild type	Yes
CRC5	KRAS G12, G13	p.G12S, VAF = 14%	Wild type	Yes, VAF = 8%
**Other clinically relevant mutations **				
**Sample**	**Mutation**	**Illumina**	**Nanopore**	**Notes**
CRC1	BRAF p.V600E	p.V600E, VAF = 24%	Wild type	Detected by targeted clinical test (VAF = 38%)
CRC4	KRAS p.Q61H	p.Q61H, VAF = 45%	Wild type	-

## Data Availability

Due to privacy issues, we do not provide the full data availability regarding the single-cell sequencing part of this publication. The data are used according to the consent provided by participants and do not compromise their anonymity. Upon request, we can provide availability to the data with permission for peer review purposes. As we used the results of the bulk and cfDNA sequencing of a previous publication, these are available upon permission request at https://cbioportal.vo.elte.hu/cbioportal (accessed on 19 September 2022).

## Appendix A

Non-biological variation in sequencing data is often due to technical factors such as fluctuations in laboratory conditions, differences in handling techniques, the variability of the individual technician, or the timing of the experiment. In our high-coverage study, all samples were processed using a standardized protocol by the same technician under consistent laboratory conditions. However, since one of the three samples was prepared and sequenced several months before the others, it was necessary to assess the possible batch effects. To address this, we performed a two-part analysis: first, we compared sequencing metrics across the original and additional flow cell datasets; second, we completed a principal component analysis (PCA) to identify any batch-related variation in the sequencing variant data.

In summary, we compiled key sequencing metrics including coverage, nucleotide base composition (A, T, G, and C), and mapping quality, which were used to calculate mapping error and accuracy. Since the PCA analysis was conducted on variant data from different sequencing runs of the same sample, we also included general variant characteristics alongside the base-level statistics. All of these metrics are presented in Table A1.

**Table A1:** Comparison of sequencing metrics across original and additional flow cell datasets for samples CRC1 and CRC3.

Metric	Original Data		Additional Flow Cell 1		Additional Flow Cell 2	
Sample ID	CRC1	CRC3	CRC1	CRC3	CRC1	CRC3
Coverage	24.77	22.64	33.48	21.35	30.82	19.43
A (%)	29.29	29.17	29.19	28.65	29.15	28.62
T (%)	29.31	29.18	29.24	28.68	29.24	28.65
G (%)	20.67	20.81	20.75	21.30	20.76	21.33
C (%)	20.72	20.84	20.82	21.38	20.85	21.39
Mapping Quality	28.5	26.7	34.6	33.9	34.8	33.9
Mapping Error (%)	4.66	4.69	0.03	0.04	0.03	0.04
Mapping Accuracy (%)	95.34	95.31	99.97	99.96	99.97	99.96
SNVs	4,425,331	4,225,383	4,579,109	4,248,173	4,562,410	4,250,886
Indels	758,668	723,412	765,947	734,694	765,366	737,695
**Germline Mutations**						
Total Variants	5,249,716	5,003,320	5,377,848	5,013,894	5,364,418	5,023,767
SNP	5,180,696	4,946,472	5,340,741	4,982,867	5,327,776	4,988,581
SV	34,510	28,424	37,107	31,027	36,642	35,186
Deletion	15,497	12,971	16,343	13,714	16,105	15,516
Insertion	18,670	15,184	20,294	17,004	20,112	19,237
Duplication	16	16	41	26	27	49
Breakend Pairs	327	253	429	283	398	384

Principal component analysis (PCA) revealed a clear separation between BatchA (Run1) and BatchB (Run2) along the first two principal components, highlighting a pronounced batch effect (Figure A1a). This pattern indicates that the variation in genotype data is predominantly driven by technical factors—such as differences in sequencing runs or laboratory conditions—rather than true biological differences. To address this issue and minimize confounding in downstream analyses, we applied the ComBat correction method, which adjusts for batch effects while preserving biological signals. As shown in Figure A1b, ComBat successfully eliminated the batch-driven variation, resulting in a unified distribution of samples across batches.

## Appendix B

**Table A2:** Methylation and modified base percentages in samples with and without PCR amplification.

Methylation, Modified Bases	Without PCR Amplification		With PCR Amplification	
	tr4	tr6	tr4	tr6
5mC	67.00%	59.00%	0.087%	0.084%
5hmC	5.00%	4.00%	0.024%	0.020%
C	28.00%	37.00%	98.87%	98.93%
